# Wearable Sensor-Based Human Activity Recognition via Two-Layer Diversity-Enhanced Multiclassifier Recognition Method

**DOI:** 10.3390/s19092039

**Published:** 2019-04-30

**Authors:** Yiming Tian, Xitai Wang, Lingling Chen, Zuojun Liu

**Affiliations:** 1 School of Artificial Intelligence, Hebei University of Technology, Tianjin 300130, China; xitaiwang56@gmail.com (X.W.); chenling@hebut.edu.cn (L.C.); liuzuojun@hebut.edu.cn (Z.L.); 2 National Research Center for Rehabilitation Technical Aids, Beijing Key Laboratory of Rehabilitation Technical Aids for Old-Age Disability, Key Laboratory of Human Motion Analysis and Rehabilitation Technology of the Ministry of Civil Affairs, Beijing 100176, China

**Keywords:** activity recognition, wearable sensor, kernel Fisher discriminant analysis, classifier ensembles, multiclassifier design and evaluation

## Abstract

Sensor-based human activity recognition can benefit a variety of applications such as health care, fitness, smart homes, rehabilitation training, and so forth. In this paper, we propose a novel two-layer diversity-enhanced multiclassifier recognition method for single wearable accelerometer-based human activity recognition, which contains data-based and classifier-based diversity enhancement. Firstly, we introduce the kernel Fisher discriminant analysis (KFDA) technique to spatially transform the training samples and enhance the discrimination between activities. In addition, bootstrap resampling is utilized to increase the diversities of the dataset for training the base classifiers in the multiclassifier system. Secondly, a combined diversity measure for selecting the base classifiers with excellent performance and large diversity is proposed to optimize the performance of the multiclassifier system. Lastly, majority voting is utilized to combine the preferred base classifiers. Experiments showed that the data-based diversity enhancement can improve the discriminance of different activity samples and promote the generation of base classifiers with different structures and performances. Compared with random selection and traditional ensemble methods, including Bagging and Adaboost, the proposed method achieved 92.3% accuracy and 90.7% recall, which demonstrates better performance in activity recognition.

## 1. Introduction 

Human activity recognition (HAR), as a new research area in the field of pattern recognition, has become a topic of focus for many scholars. During the past decade, HAR, especially the activities of daily living (ADL) such as walking, sitting, lying, jumping, and so forth, has attracted much attention from researchers worldwide. Various HAR systems have been proposed by researchers as a medium to obtain additional information about people’s activities. By analyzing the information from patients’ activities, doctors have been able to diagnose some chronic diseases [1] as well as develop rehabilitation plans for Parkinson’s patients [2]. Thus, HAR can provide the elderly with better-quality healthcare. Moreover, HAR is also important for applications including human–computer interaction, surveillance, keeping track of athletic activities [3], and so on.

There have been many solutions to HAR. These can be roughly divided into three aspects: video-based, environment interactive sensor-based, and wearable sensor-based solutions [4]. For video-based solutions, thermal cameras and depth cameras such as Microsoft Kinect and Intel SR300 have been utilized in activity recognition and experienced great breakthroughs. For example, Xia et al. found that it was feasible to use the Kinect for respiratory motion tracking [5]. Qin et al. presented a novel method for real-time markerless hand gesture recognition from depth images [6]. In [7], thermal and depth cameras have been utilized for multimodal detection of breathing patterns. A review article on both handcrafted and deep-learning-based action representations for vision-based HAR was presented in [8]. It compared these two approaches and presented the well-known public datasets available for experimentation. In general, video-based solutions perform well if, in a well-controlled environment, it is especially suitable for security (e.g, intrusion detection) and some tracking applications. One problem of video-based solutions is that people’s privacy may be violated if the cameras are installed in some places, such as bathrooms and bedrooms, which limits their application area. Moreover, the performance of video-based solutions may be not robust and reliable if there is clutter or variable lighting in the environment. Last, but not least, video-based solutions are relatively expensive [9,10]. Environment interactive sensor-based solutions would not violate the subject’s privacy and are suitable for recognizing daily living activities in rooms. However, this approach is generally costly due to the numerous sensors deployed in appropriate places and is often limited to indoor scenarios [11,12]. This fact hinders such a real-time HAR system from being scalable.

With the current development of the microelectronics, miniature and flexible sensors such as accelerometers, gyroscopes, proximity sensors, humidity sensors, and so forth, bring convenience to the users [13,14,15,16]. Compared with video-based solutions, the wearable sensor-based approach has the advantage of being light and compact, which allows it to collect people’s motion information all the time and anywhere. This kind of approach is also suitable for both indoor and outdoor environments. Therefore, the wearable sensor-based approach can be a good candidate for human activity recognition.

Most HAR studies have utilized sensors from multiple body positions to collect human activity information for recognition [17,18,19]. These systems achieve good recognition results with indistinguishable activities. However, they are not suitable for long-term applications because multiple sensors can cause inconvenience to users. Comparatively, a small number of studies have utilized a single sensor attached to a subject’s body part, such as the waist, chest, or ankle, to collect activity information [20,21,22,23]. This approach is suitable for long-term activity monitoring and achieves good recognition results for some basic activities such as lying, walking, and running. However, it is not reliable when dealing with complex and similar activity types such as going downstairs and upstairs. Moreover, it may work even worse when processing different data recordings of similar activity caused by individual differences [24,25]. Despite significant research efforts to find out the most effective feature selection and feature transformation methods for single-sensor-based HAR, improvements in the robustness and generalization of activity recognition problems with large data variations are still very limited.

As a pattern recognition problem, two aspects could make HAR challenging. First, different subject-related features such as gender, age, weight, and height make HAR a complex problem. For example, the adult and the elder do not have the same kind of data when they are walking or running. Second, the variety of styles with which people perform a certain activity under different external environments is another challenge [26,27]. These problems require the recognition system to not only have good recognition accuracy but to also have good generalization ability. Among the methods that can improve the generalization and robustness performance of recognition systems, combinations of multiple classifiers have been demonstrated to be very effective [28,29,30]. However, due to the redundancy between the base classifiers, there is no guarantee that there will be a good complementary relationship between all base classifiers, and some basic classifiers may not contribute to improving system performance. Selecting base classifiers with excellent performance and complementarity can further improve the performance of the recognition system [31,32,33]. This approach is similar to optimizing feature sets to reduce feature dimensions and obtain a more robust feature set. However, as we know, there are very few studies that have applied this classifier approach for constructing a recognition system in HAR. 

To address these issues, in this paper, we present a two-layer diversity-enhanced multiclassifier recognition method. Since accelerometers are commonly used and have been proven to be effective for human activity recognition [29,34], we only used accelerometer data in this study. Three kinds of features, including time-domain features, autoregressive (AR) coefficients and frequency-domain features, were extracted from a sliding window of data. During the experiments, we first investigated the performance analysis of the effect of training set diversity enhancement on activities that are easily misrecognized using the whole set of base classifiers. Then, we explored the structure and performance of each base classifier in the whole set of base classifiers. Last, we validated the effectiveness of our proposed classifier selection method in activity recognition through a number of comparative experiments.

The main contributions of this paper are summarized as follows:

(1) A novel multiclassifier recognition framework that considers training set and base classifier diversity was proposed to enhance the generalization performance of the HAR system and improve the system’s adaptability to different individuals.

(2) A kernel Fisher discriminant analysis (KFDA) was performed to process the extracted features to enhance the discrimination between different activities, and bootstrap resampling was utilized to increase the generalization performance of classifiers by creating diverse training sets.

(3) A classifier selection approach was applied to the field of activity recognition for the first time, and a novel classifier selection method was proposed to optimize the performance of a multiclassifier recognition system.

(4) We demonstrated that the proposed method is reliable and accurate for activity recognition by collecting sensor data from subjects with a great diversity of subject-related features and comparing the performance of the proposed method to some traditional ensemble methods.

The paper is organized as follows: In Section 2, related works that focus on feature selection and multiclassifier recognition systems in activity recognition are introduced. In Section 3, we present details of the proposed activity recognition approach. Following that, Section 4 introduces the experiment and results. Finally, we draw conclusions in Section 5.

## 2. Related Work

The fundamental problem of recognition algorithm design is how to improve the generalization ability and robustness of the recognition system. The performance of the recognition system can be improved by integrating multiple learning individuals that meet certain conditions. Many researchers currently utilize multiclassifier schemes to improve the accuracy of HAR. Catal et al. [29] established an activity recognition model based on a machine learning classifier. It combines the J48, multilayer perceptron (MLP), and logistic classifiers using the mean method, and the average recognition rate of the model is 97%. Lee et al. [35] proposed a hybrid expert model based on a smart device to recognize human activity that had a recognition accuracy of up to 92.56%. Yuan et al. [36] utilized the output of multiple speed learning machines to perform simple mean algorithm fusion processing, and the final model output recognition accuracy was 6% higher than that of a single speed learning machine. Cao et al. [37] optimized the deployment of multisensors by pruning the multiple ensemble classifier. Through the proposed method, the number and type of multisensor can be appropriately decided. Bayat et al. [38] built an ensemble-learning-based HAR model which contained three classifiers: MLP, SVM (Support Vector Machine) and LogitBoost for in-hand phone position. This model achieved 91.15% accuracy when recognizing six activities. The experiments also showed that the average of the probabilities was better than majority voting as a fusion method for the proposed model. An ensemble model was built in [39] using AdaBoost in combination with the decision tree algorithm C4.5 and other base classifiers. The study found that the AdaBoost–C4.5 ensemble model achieved a higher overall accuracy level of 94.04%. Although the above studies have improved the recognition accuracy of the classification system, the individual differences between the classifiers have not been considered. The classifiers participating in the multiclassifier should not only satisfy the accuracy but also must have certain diversities.

Some advanced works have concentrated on feature studies in HAR. Ronao et al. [40] applied data mining technology to mobile phone sensor-based activity recognition and a deep convolutional neural network was utilized as an automatic feature extractor and classifier. However, this method requires a relatively large data processing capability for the hardware device. Some studies utilized feature transformation methods to reduce the feature dimension while also enhancing the distinguishing ability of feature vectors. For example, [22,41] introduced linear discriminant analysis (LDA) to enhance the discrimination between different activities and make features more robust to be useful for fast activity recognition. In order to reduce the influence of the sensor’s varying locations and orientations on the recognition performance, principal component analysis (PCA) was employed in [42] to realize location-adaptive activity recognition. Wang et al. [43] proposed a hybrid feature selection method to reduce feature dimensions. This method combined the traditional feature selection method filter and wrapper. The experimental results showed that the method fully balances the relationship between recognition efficiency and accuracy. Motivated by the success of the weightlessness feature, Tao et al. [44] proposed a new two-directional feature for bidirectional long short-term memory (BLSTM) for incremental learning in human activity recognition. Experiments on the naturalistic mobile-device-based human activity dataset suggested that it is superior to other methods. Forster et al. [45] proposed a feature extraction method based on genetic programming to obtain a feature set that is robust to sensor position. Experiments on a fitness activity dataset showed that the method achieved an accuracy of 73.4% in contrast to 70.1% when using one selected standard feature. Wang et al. [46] presented a game-theory-based feature selection method to select distinguished features and reduce computational cost. The experiments showed that the proposed method performed better when compared with ReliefF and mRMR.

Table 1 summarizes these activity recognition studies using wearable sensors on multiclassifier schemes and features.

## 3. The Proposed Framework

**Figure 1:**
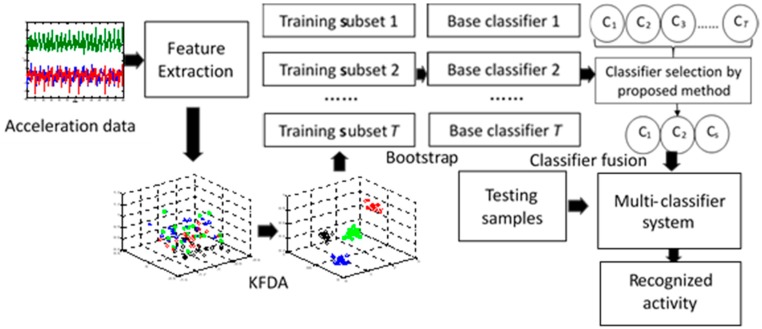
Workflow of the proposed activity recognition approach.

Figure 1 shows the workflow of the proposed activity recognition approach. Briefly, the dataset used in this paper was acquired in our laboratory and the acceleration in three axes from the TrignoTM wireless system was utilized for the experiment. The approach involved three main modules. First, after feature extraction from the acceleration in three axes, KFDA was utilized to increase the discrimination of different activities. This can improve the recognition accuracy of some similar activities. Second, a bootstrap technique was utilized to create randomized training data after applying KFDA to transform features, which can improve the generalization performance of the recognition system and make it more suitable for dealing with subjects with larger diversity. These two steps can be considered as the data-based first layer of diversity enhancement. Third, the classifier selection approach based on combined diversity measures was proposed to optimize the multiclassifier system. The base classifiers that perform better and with more diversities were selected and combined to recognize activities. The third step can be considered as the classifier-based second layer of diversity enhancement. In the following subsections, we describe the details of these modules.

### 3.1. Feature Extraction

**Figure 2:**
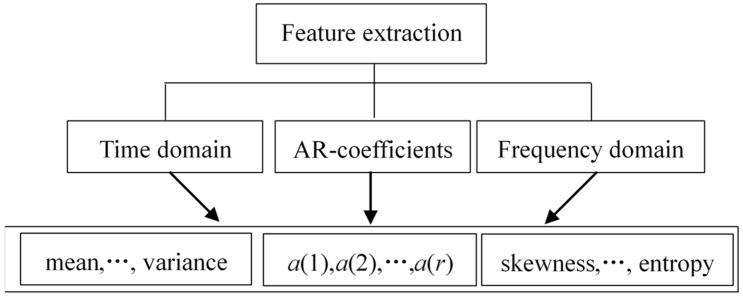
Block diagram for feature extraction in our activity recognition approach.

After denoising the signal, we used the sliding window technique to segment the acceleration signal. Then, features were extracted from each sliding window of 300 samples with 50% overlapping between consecutive windows. The sampling rate was 150 Hz. These kinds of features include time-domain features, AR coefficients, and frequency-domain features. For each sliding window, we calculated the mean value, standard deviation, maximal value, minimal value, median absolute deviation, signal magnitude area, and interquartile range as the time-domain features. The model order of the AR coefficients was set to *P* = 3 based on [46] in this research; thus, a total of nine AR coefficients were obtained in each sliding window. In the frequency domain, we applied the fast Fourier transform (FFT) algorithm to extract the frequency features, which included mean value, skewness, kurtosis energy, and entropy. The effectiveness of these features for HAR have been proved by many reseasrch works [46,47,48]. The block diagram of this module is shown in Figure 2.

### 3.2. Kernel Fisher Discriminant Analysis (KFDA)

Fisher discriminant analysis (FDA) is a feature dimension reduction and classification method developed in the field of pattern recognition. Its core idea is projecting test data into a certain direction according to different characteristics of the sample and maximizing the interclass dispersion of the projection of the test data while minimizing intraclass dispersion. Although the traditional Fisher discriminant has been widely used in activity recognition, when the nonlinearity between variables is serious, it is difficult to find a suitable direction to maximize the separation of the projection of test data. For nonlinear classification, the introduction of the kernel function method into Fisher discriminant analysis has achieved good results. Mika et al. [49] first proposed KFDA, which is briefly described below.

Suppose that all sample points in the *p*-dimensional space have *C* classes: *G*_1_, *G*_2_, ..., *G_C_*, and the total number of samples is *N*. The *j*th (*j* = 1, 2, ..., *C*) classes *G_j_* contain *N_j_* samples written as $x_{j,}^{1}\;x_{j}^{2},\;\cdots ,\;x_{j}^{Nj}$.

The sample *x*∈*R^p^* passes through the nonlinear high-dimensional mapping φ and the corresponding mode φ(*x*)∈H. In the high-dimensional feature space *H*, the intraclass dispersion ***S***_W_ and the interclass dispersion ***S***_B_ of the training samples are, respectively,

(1) $$S_{W}=\frac{1}{N}\sum_{i=1}^{C}\sum_{j=1}^{N_{i}}\left[\phi (x_{i}^{j})-m_{i}\right]{\left[\phi (x_{i}^{j})-m_{i}\right]}^{T}$$

(2) $$S_{B}=\frac{1}{N}\sum_{i=1}^{C}N_{i}(m_{i}-m){\left(m_{i}-m\right)}^{T}$$

where ***m**_i_* represents the *i*th sample mean in the feature space *H*: $m_{i}=(1/N_{i})\sum_{j=1}^{N_{i}}\phi (x_{j})$, and ***m*** represents the mean of all sample points in the feature space *H*: $m_{i}=(1/N_{i})\sum_{i=1}^{C}\sum_{j=1}^{N_{i}}\phi (x_{i}^{j})$. In the feature space *H*, the Fisher criterion is

(3) $$J(w)=\max\frac{w^{T}S_{B}w}{w^{T}S_{W}w}$$

where ***w*** is any nonzero column vector. The Fisher discriminant finds the best projection vector ***w*** by optimizing Equation (3). Since the feature space *H* dimension is too high, ***w*** cannot be directly obtained, thus introducing a kernel function.

(4) k(x,z)=〈ϕ(x),ϕ(z)〉

Equation (4) indicates that any two inner product vectors in the high-dimensional space *H* can be represented by a kernel function. Then, the optimal solution ***w*** in Equation (3) can be expressed as $w=\sum_{i=1}^{N}\alpha_{i}\phi (x_{i})$, where ***α*** = (*α*_1_, *α*_2_, ..., *α_N_*)^T^ is a column of vectors, so in the high-dimensional feature space *H*, the Fisher criterion becomes

(5) $$J(\alpha )=\max\frac{w^{T}S_{B}w}{w^{T}S_{W}w}=\max\frac{\alpha^{T}K_{B}\alpha }{\alpha^{T}K_{W}\alpha }$$

In the formula, ***K***_B_ and ***K***_W_ are calculated as follows:

$$\left\{ \begin{array}{l} K_{B}=\frac{1}{C(C-1)}\sum_{i=1}^{C}\sum_{j=1}^{C}(\mu_{i}-\mu_{j}){\left(\mu_{i}-\mu_{j}\right)}^{T}\\ \mu_{i}={\left[\frac{1}{N_{i}}\sum_{j=1}^{N_{i}}k(x_{1},x_{i}^{j}),\cdots ,\frac{1}{N_{i}}\sum_{j=1}^{N_{i}}k(x_{N},x_{i}^{j})\right]}^{T} \end{array}\right.$$

$$\left\{ \begin{array}{l} K_{W}=\frac{1}{C}\sum_{i=1}^{C}\frac{1}{N_{i}}\sum_{j=1}^{N_{i}}(\xi_{j}-\mu_{j}){\left(\xi_{j}-\mu_{j}\right)}^{T}\\ \xi_{j}={\left(k(x_{1},x_{j}),k(x_{2},x_{j}),\cdots ,k(x_{N},x_{j})\right)}^{T} \end{array}\right.$$

Therefore, the problem of Equation (5) is transformed into maximizing $K_{W}^{-1}K_{B}$ and its corresponding eigenvector. In practical applications, ***K***_W_ is often not guaranteed to be nonsingular. Therefore, ***K***_W_ + ***σI*** is often used to replace ***K***_W_, where ***σ*** is a positive number and is usually ***σ*** = 10^−7^, and ***I*** is the identity matrix.

In the kernel Fisher discriminant method, the selection of the kernel function is very important. There are many kinds of kernel functions, and the most common ones are the linear kernel function, polynomial kernel function, radial basis function, and sigmoid kernel function. However, there is no good method for constructing an optimal kernel, and there is no deep theoretical research. In the literature [50], through experimental research, it was found that the radial basis function (RBF) has better classification ability under the default parameters. In this study, the RBF kernel function was used:

(6) $$k(x,z)=\exp(-\frac{{\left\|x-z\right\|}^{2}}{\delta^{2}})$$

**Figure 3:**
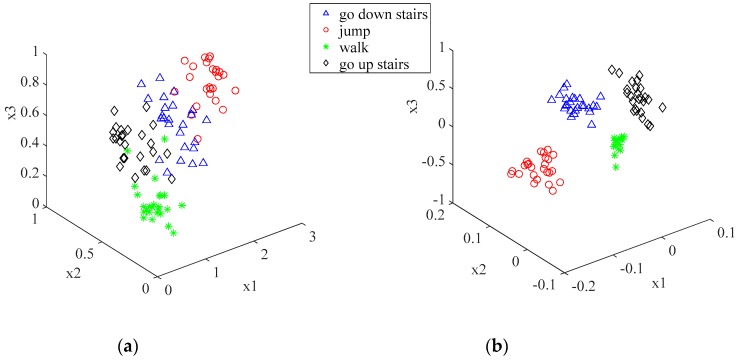
Features without and with kernel Fisher discriminant analysis (KFDA) operations. (**a**) 3D feature space representation on original features (x1 is the mean value of the *y*-axis, x2 is the standard deviation of the *x*-axis, x3 is the standard deviation of the *y*-axis); (**b**) 3D space representation of the first three KFDA-based features.

In the formula, the parameter *δ* is positive, and the selection of *δ* is an optimization problem. In this study, the cross-validation method was used to select the parameter *δ*. The 3D feature plots for the four activities before and after applying the KFDA are shown in Figure 3.

### 3.3. Bootstrap Resampling 

**Figure 4:**
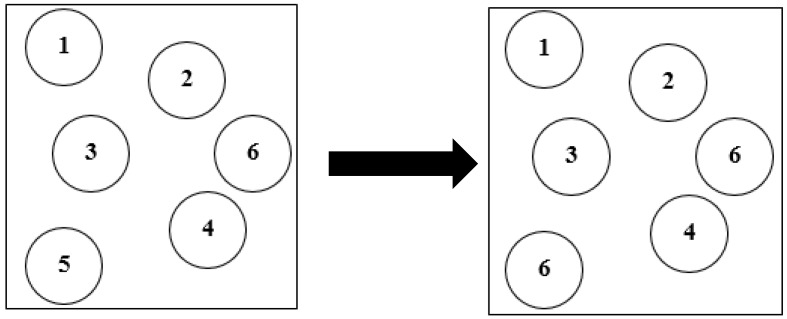
Dataset after being processed by bootstrap resampling.

Bootstrap resampling or the bootstrap technique is a uniform sampling method that is put back from a given training set. Each time a sample is selected, it may be selected again and added to the training set. Obtaining samples by this method is simple, convenient, and easy to implement [51]. In the ensemble classifier, diversity (classifiers should be independent, that is, containing uncorrelated errors) between base classifiers is a very important condition for constructing an ensemble classifier. A number of studies have proved that utilizing bootstrap resampled data to train base classifiers can improve the generalization of a recognition system, such as the bootstrap aggregation neural networks proposed by Zhang [52]. Unlike these works, our study not only utilized the bootstrap technique to obtain different training subsets from the original samples but also considered the diversities of base classifiers. Therefore, a diversity-measure-based classifier selection approach was proposed to further increase the diversity of base classifiers. Figure 4 illustrates bootstrap resampling with replacement. In this particular realization, data sample 6 was sampled twice, but data sample 5 was not sampled.

### 3.4. Classification Algorithm

**Figure 5:**
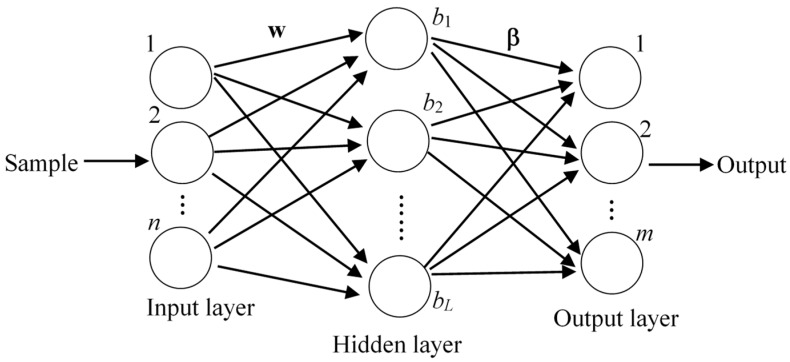
The structure of single hidden layer feed-forward neural network.

This research utilized the extreme learning machine (ELM) as a base classifier since it is widely used in sensor-based activity recognition research and many studies have demonstrated its good generalization ability [53,54]. As a single hidden layer feed-forward neuron network (SLFN), the input weights and biases of ELM can be randomly selected. Figure 5 shows the structure of ELM. In this study, each neuron in the input layer corresponded to one feature in the feature set and *m* neurons in the output layer, respectively, corresponded to the activities to be recognized.

For any *N* different samples (**X***_j_*, **t***_j_*), *j* = 1,2,…*N*, where $x_{j}={\left[x_{j1},x_{j2}\cdots x_{jn}\right]}^{T}$ is the *j*th sample, each sample contains *n*-dimensional features, and $t_{j}={\left[t_{j1},t_{j2},\cdots t_{jm}\right]}^{T}$ is the encoded class label. All samples belong to *m* different activities, and the ELM mathematical model with *L* hidden neurons can be expressed as

(7) $$\sum_{i=1}^{L}\beta_{i}g(w_{i}\cdot x_{j}+b_{i})=t_{j},\;j=1,\cdots N$$

where *g*(*x*) is the excitation function; in this paper, the softmax excitation function was used. **w***_i_*, *b_i_*, and **β***_i_* are the input weight, hidden element offset, and output weights of the *i*th hidden neuron node, respectively. The input weights and hidden element offsets were randomly initialized from the range of [−1, +1]. Equation (7) can be written in matrix form:

(8) Hβ=T

where **β** represents the output weight, **T** is the corresponding coding class label, and **H** is the hidden layer output matrix:

(9) $$H={\left[\begin{array}{ccc} g(w_{1}\cdot x_{1}+b_{1}) & \cdots & g(w_{L}\cdot x_{j}+b_{L})\\ \vdots & \cdots & \vdots \\ g(w_{1}\cdot x_{N}+b_{1}) & \cdots & g(w_{L}\cdot x_{N}+b_{L}) \end{array}\right]}_{N\times L}$$

Since the neural network system is linear, the **β** output weight is obtained by the following equation:

(10) $$\beta =H^{†}T$$

where **H**^†^ is the generalized inverse matrix of **H**. 

### 3.5. Diversity Measures and the Proposed Classifier Selection Method

**Figure 6:**
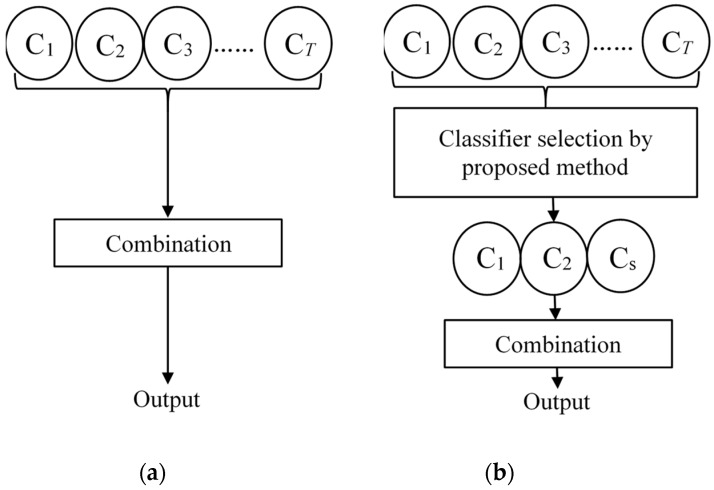
Comparison of the combination method with classifier selection or not: (**a**) direct combination method and (**b**) selection-based combination method.

The main aim of multiple classifiers is to improve the generalization capability and classification performance of the recognition system. Most studies have combined all the trained base classifiers without considering the relationship of the base classifiers. However, there may be poor performance and redundant base classifiers affecting the performance of the recognition system. Therefore, in this research, our goal was not to utilize all base classifiers to establish an ensemble recognition system but to perform a performance and diversity analysis using the proposed classifier selection method on the base classifier and then selectively combine the base classifiers. Figure 6 shows the comparison of a direct combination method and a selection-based combination method. The diversity measures we utilized in this research are described below.

#### 3.5.1. Diversity Measures 

##### Disagreement Measure

Skalak proposed the disagreement measure from the concept of diversity [55]. The larger the disagreement measure, the greater the diversity between the base classifiers. The disagreement measure for the two base classifiers *C_i_* and *C_k_* can be expressed by the following formula:

(11) $${Dis}_{ik}=\frac{N^{01}+N^{10}}{N^{11}+N^{10}+N^{01}+N^{00}}$$

where *N*^00^ represents the proportion of samples when classifiers *C_i_ and C_k_* recognize errors, *N*^11^ represents the proportion of samples when classifiers *C_i_* and *C_k_* recognize correctly, *N*^10^ represents the proportion of samples when classifier *C_i_* recognizes errors while classifier *C_k_* recognizes correctly, and *N*^01^ represents the proportion of samples when classifier *C_i_* recognizes correctly while classifier *C_k_* recognizes errors. The formula of disagreement measure extended to *L* classifiers can be expressed as

(12) $${Dis}_{av}=\frac{2}{L(L-1)}\sum_{i=1}^{L-1}\sum_{k=i+1}^{L}{Dis}_{ik}$$

##### Cunningham’s Entropy

“Entropy” was originally a physical quantity in information theory that was used to measure the uncertainty of the source. Cunningham et al. [56] extended this concept to apply it to the diversity measure of classifiers:

(13) $$Ent=\frac{1}{N}\sum_{i=1}^{N}\frac{1}{L-\left\lfloor \frac{L}{2}\right\rfloor -1}\min(l(x_{i}),L-l(x_{i}))$$

where *N* is the number of test samples, *L* is the number of base classifiers, and *l*(*x_i_*) represents the number of base classifiers that correctly classify sample *x_i_*. *Ent*∈[0, 1], and the value of *Ent* is proportional to the diversity between the base classifiers. 

##### Coincident Failure Diversity

Partridge et al. [57] proposed the principle of generalized diversity and consistent failure diversity for non-one-to-one diversity metrics. The latter is more commonly used in the corresponding research. The definition of consistent failure diversity is as follows:

(14) $$CFD=\left\{ \begin{array}{ll} 0, & P_{0}=1,0\\ \frac{1}{1-P_{0}}\sum_{i=1}^{L}\frac{L-i}{L-1}P_{i} & P_{0}<1 \end{array}\right.$$

where *P*_0_ represents the probability that all the base classifiers are correctly classified, and *P_i_* represents the probability that the *i*th base classifiers in the *L* base classifiers that make up the ensemble classifier are classified correctly. The larger the value of *CFD*, the greater the diversity between the base classifiers. 

#### 3.5.2. The Proposed Classifier Selection Method

**Figure 7:**
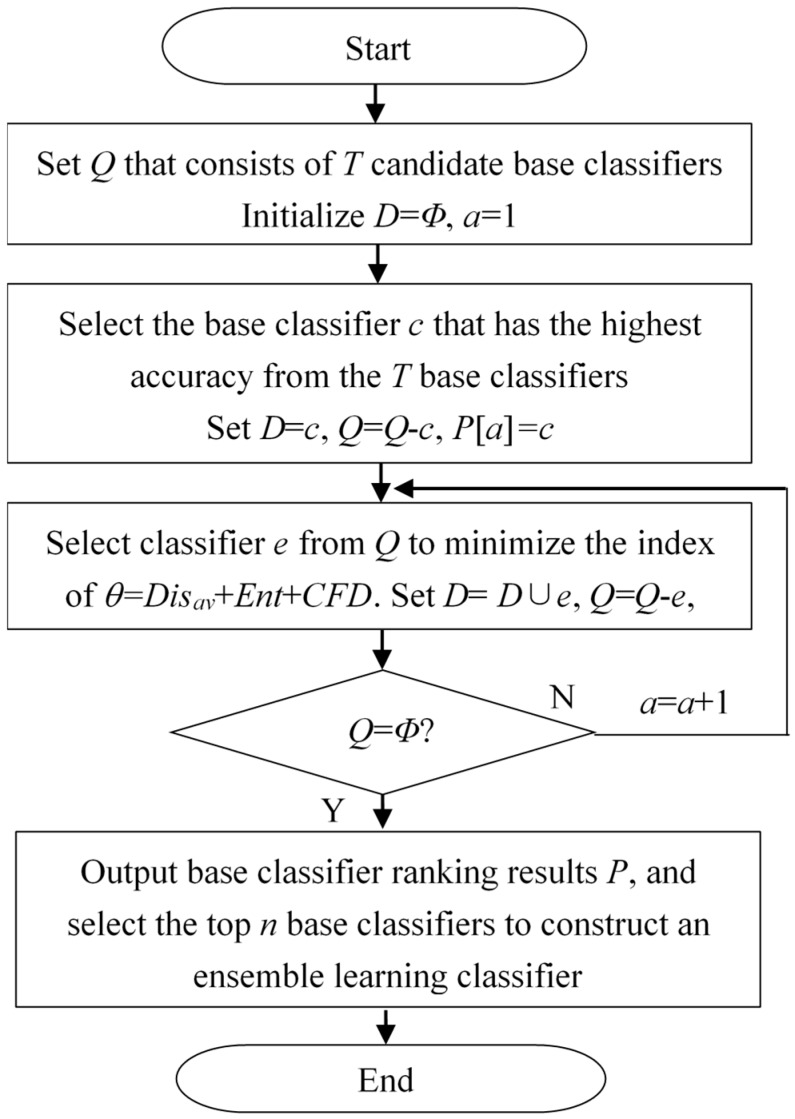
Flowchart of the proposed classifier selection method.

Similar to feature selection, the selection of base classifiers can eliminate redundant or poorly performing classifiers, reduce computational burden, and optimize the relationship between base classifiers in a multiclassifier recognition system. Unlike most studies on selecting base classifiers based on only one diversity measure, the proposed classifier selection method proposed in this paper was based on the above three diversity measurement methods. The accuracy rate and the diversity of base classifiers were considered as the two selection principles. Based on these two principles, we ranked all the base classifiers and selected the top *n* base classifiers to construct a multiclassifier recognition system. Figure 7 shows the flowchart of the proposed classifier selection method. The experiments were implemented in Matlab 2012a.

The proposed base classifier selection procedure can be summarized as follows:

Step 1: The *T* ensemble base classifiers are supervised trained with different training data subsets. 

Step 2: The validation dataset is utilized to find out the base classifier *c* with the highest recognition accuracy among all the base classifiers and put base classifier *c* in set *D* and rank first.

Step 3: The base classifier *e* is selected from the remaining candidate base classifier set *T*. The base classifier *e* should satisfy the criterion of minimizing the index of *θ = Dis_av_* + *ENT* + *CFD* with the previously selected base classifier (in set *D*). Then, put base classifier *c* in set *D* and rank second.

Step 4: Then, select base classifier *e* repeatedly until all base classifiers are selected into set *D*. The order in which each classifier is selected is the ranking result.

Step 5: According to the ranking result *P*, select the top *n* base classifiers to construct a recognition system by majority vote.

## 4. Experimental Results and Analysis

### 4.1. Experimental Setup and Experimental Dataset

**Figure 8:**
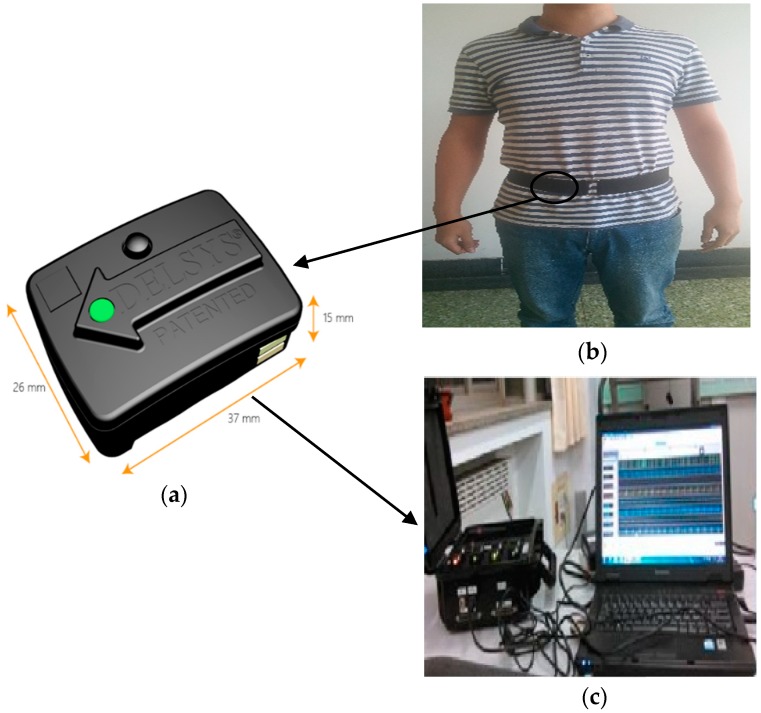
The experimental platform and sensor placement: (**a**) signal acquisition device, (**b**) the placements of the collection node, and (**c**) the process of data collection.

**Figure 9:**
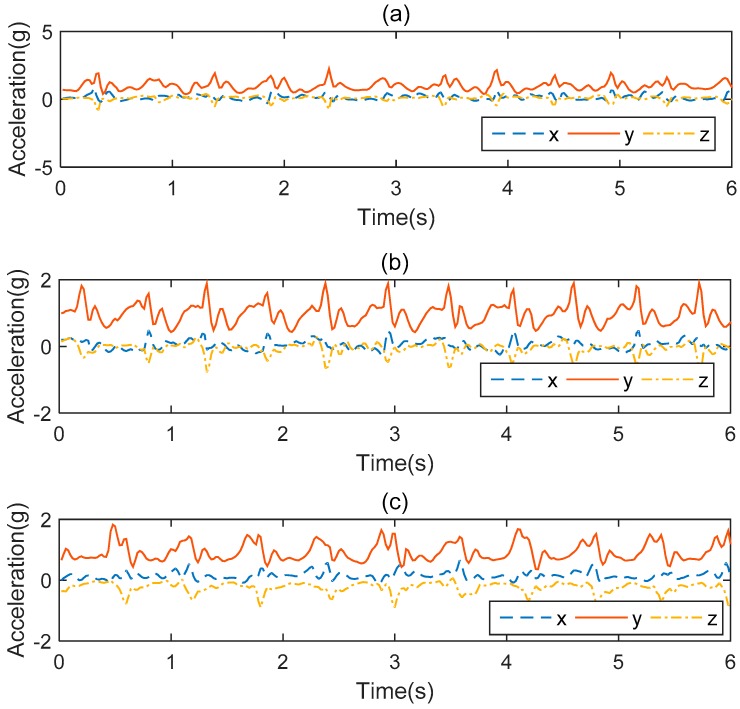
The triaxial accelerometer data of “go up stairs” from three subjects with large individual differences: (**a**) Male, 177 cm, 83 kg; (**b**) Female, 162 cm, 45 kg; (**c**) Male, 172 cm, 60 kg.

In the experiment, we used a TrignoTM wireless electromyogram (EMG) acquisition instrument produced by Delsys Company as the activity information collection equipment, as shown in Figure 8a. TrignoTM is a high-performance, high-precision biosignal acquisition device which integrates the data acquisition function of myoelectricity and acceleration. It has been widely used in medicine, sports, and engineering. In this study, we only used the inertial information of human activity acquired by its accelerometer. The acceleration was accurate to ±6 *G* with a resolution of 0.016 (*G* is the gravitational constant), and the accelerometer worked at a sampling rate of 150 Hz. In the data collection process, the effects of individual differences such as age, height, and weight on the signal distribution were taken into account. Therefore, 10 subjects (5 male, 5 female) with different physical characteristics were selected to participate in the experiment. Their age, height, and weight statistics are shown in Table 2. To capture day-to-day variations and signals in different environments, each subject was required to wear a sensor at the same position on the waist (as shown in Figure 8b) and perform five trials for each activity on different days and in environments. Figure 8c shows the process of data collection. Before the start of each experiment, we checked that the sensor was fixed in the same position as the previous subject and a strap was used to secure the sensor to the waist to prevent movement during the collection process. Each subject followed the sequence of the activity numbers in the left column of Table 3 and performed each activity for about 20 s. The total time of each activity is listed in the right column of Table 3. We expected that by doing this, the activities performed by the subjects would be more similar to their styles in real life and we would be better able to verify the performance of our proposed method. Figure 9 shows the acceleration signals from three subjects with large individual differences as they went up stairs. It can be found from Figure 9 that individual differences had a large influence on the amplitude and trend of the acceleration signal. We used the leave-one-out (LOO) strategy to evaluate the proposed method. The verification was repeated 10 times and each dataset from a subject was used once for testing. We obtained the final result by calculating their average.

### 4.2. Performance Measures

This study used the performance measures accuracy and recall, which are also commonly used in the field of activity recognition. These two measures can be expressed as follows:

(15) $$Accuracy=\frac{TP+TN}{TP+TN+FP+FN}$$

(16) $$Recall=\frac{TP}{TP+FN}$$

where the variables *TP*, *TN*, *FP*, and *FN*, respectively, represent the number of true positive, true negative, false positive, and false negative outcomes in a given experiment.

### 4.3. Experimental Results

#### 4.3.1. PCA-Based Features versus FDA-Based Features versus KFDA-Based Features

**Figure 10:**
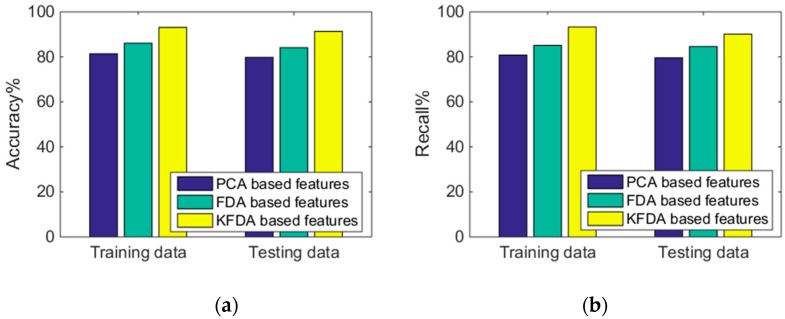
Performance comparison: (**a**) accuracy comparison of the different feature selection methods and (**b**) recall comparison of the different feature selection methods.

We analyzed the advantages of feature transformation and the effectiveness of the proposed KFDA technique for similar activities that are easily misrecognized. PCA, which is most commonly used in the field of activity recognition [9,45], was chosen as the comparative method of feature transformation. In the comparative experiment, the test dataset was utilized to analyze the activity recognition performance of three kinds of features: PCA-based, FDA-based, and KFDA-based features. Figure 10 presents the results obtained by using PCA-based features as well as the results obtained by using our proposed KFDA-based features and comparative FDA-based features. The comparative experiments were performed under the conditions of using a multiclassifier system which contained 20 base classifiers without selection. For the parameter settings of the classification algorithm, the input weights and biases for each base ELM were randomly initialized from [−1, +1] and the number of hidden neurons was 20.

It can be observed from Figure 10 that the PCA-based features had the worst performance, with 81.4% accuracy and 80.8% recall on the testing data. In terms of features with selection, FDA-based features had better performance compared with original features, with 86.1% accuracy and 84.8% recall on the testing data. KFDA-based features achieved the best performance, with 92.3% accuracy and 90.7% recall on the testing data. After applying the KFDA, the total accuracy and recall of the recognition system were significantly increased, which reflected the effectiveness of the proposed KFDA features. Similar results can be observed from the training dataset. 

Additionally, in order to gain better insight into the different performances of the three kinds of features in activity recognition and to analyze the proposed KFDA feature transformation method to enhance the feature set distinguishing ability, a corresponding confusion matrix was constructed. Table 4, Table 5 and Table 6 present the confusion matrix of activity recognition results that were obtained with PCA-based features, FDA-based features, as well as the results obtained with our proposed KFDA-based features.

According to Table 4, we can observe that when using PCA-based features, there were many misrecognitions between walk (W) and go down stairs (GD), walk (W) and run forward (R), jump (J) and go down stairs (GD), jump (J) and go up stairs (GU), run forward (R) and go up stairs (GU), and run forward (R) and walk (W). It can be observed from Table 5 that after applying FDA, the discrimination of activities increased, such as activity W to GD, activity W to R, activity J to GD, activity J to GU, and activity R to GU and GD. From Table 6, it can be seen that it was much easier for the KFDA-based features to distinguish the activity W from lie (L), activity J from stand (S), activity J from L, activity GD from L, and activity R from J. The number of activities that were difficult to recognize by PCA- and FDA-based features, such as activities between GD and W, R and W, J and R, and J and sit on a chair (SC), also decreased significantly when using KFDA. This indicates that the KFDA-based features contain more discriminant and valuable information than the original features and PCA-based features in human activity recognition.

#### 4.3.2. The Performance of Base Classifier 

**Figure 11:**
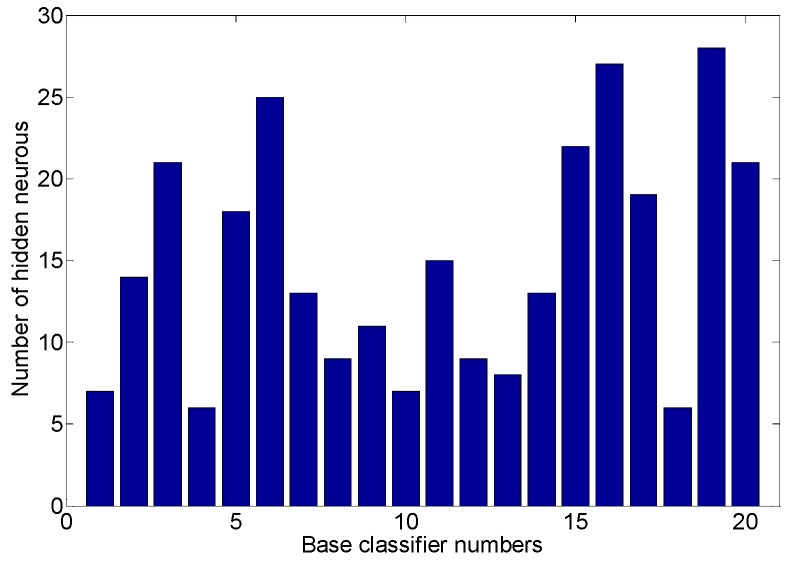
The number of hidden neurons in the base classifiers.

We analyzed the performance of each base classifier in the multiple classifier recognition system when they were trained with the bootstrap technique in the situation where the KFDA-based features were utilized. This helped us to determine the performance of the base classifier and analyze the performance correlation of the base classifier. A multiple classifier system consisting of 20 base classifiers was developed. Similar to the previous section, the input weights and biases for each base ELM were also randomly initialized from [−1, +1]. The base classifiers with different hidden neurons from [5, 30] were utilized and analyzed by experiments, and the highest accuracy was used as the base classifier hidden neuron selection criterion on the training dataset. Figure 11 shows the number of hidden neurons of base classifiers trained using the bootstrap technique. It can be seen that some base classifiers required more than 20 hidden neurons, while some base classifiers required only about 10 hidden neurons. This indicates that the structure of the “best” base classifier changed according to the training data and the base classifiers obtained by bootstrap technique training had different neural network structures.

**Figure 12:**
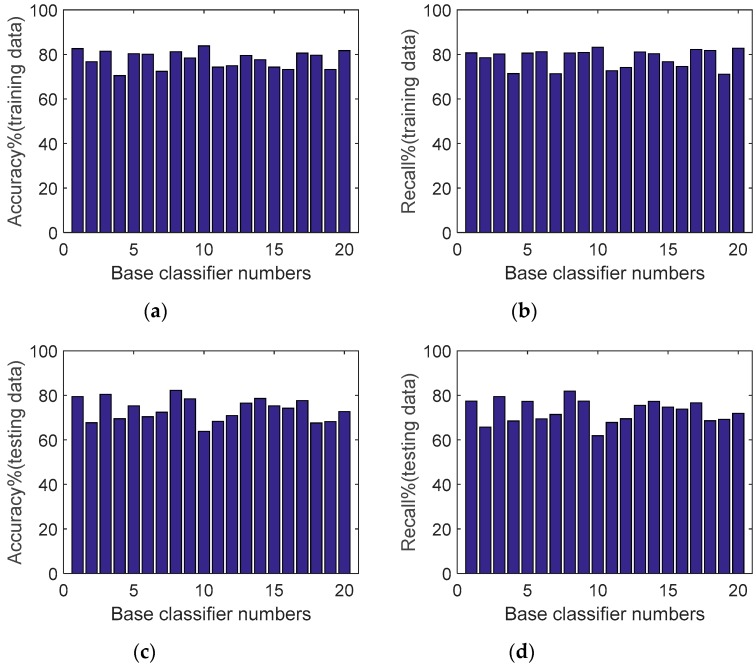
The accuracy and recall for each base classifier: (**a**) the accuracy of each base classifier in training data, (**b**) the recall of each base classifier in training data, (**c**) the accuracy of each base classifier in testing data, and (**d**) the recall of each base classifier in testing data.

Figure 12 shows the accuracy and recall values on the training and testing data from the 20 different base classifiers. As can be seen from Figure 12, the basic classifier showed inconsistent performance on the model construction data (training data) and test data, which may have been due to the large difference in data distribution between the training and test sets. For example, base classifier 10 gave the best performance on the training data, with an accuracy of 83.86% and recall of 83.26%, but its performance on the testing data was not the best. The base classifier with the best performance on the testing data was from base classifier number 8, with an accuracy of 82.2% and recall of 81.92%, but its accuracy and recall on training data were 81.2% and 80.62%, which were not optimal. Similar observations can be made for other base classifiers. This demonstrates the poor generalization performance and nonrobust nature of single classifiers when dealing with an activity dataset, especially with differences.

#### 4.3.3. The Influence of Base Classifiers with or without Selection 

The previous section experimentally proved that the base classifier trained by the bootstrap technique has different and unreliable performances on the training and testing datasets. Therefore, constructing a classifier-selection-based multiclassifier recognition system is critical to improving the performance of activity recognition. Thus, we verified the effectiveness of the proposed classifier selection method based on activity recognition through comparative experiments. First, we evaluated the diversities of the 20 base classifiers obtained and ranked the base classifiers by the method proposed in Section 3.5. Table 7 shows the results of diversities and rankings of 20 base classifiers of the multiclassifier system.

In order to verify the effectiveness of the proposed classifier selection method, we conducted several comparative experiments, including fusion of the whole set of 20 base classifiers; fusion of 15, 10, and 5 base classifiers selected by using the proposed method; and fusion of randomly selected 15, 10, and 5 base classifiers. Table 8 shows the comparison of the results from the experiment.

From Table 8, it can be found that the performance of the recognition system composed of the classifiers selected in this paper was better than that of the random selection system. This can be reflected from the performance comparison of three different sets of base classifiers. For example, when using the random selection method to fuse 15 base classifiers, the system achieved 84.68% accuracy and 84.32% recall. However, when utilizing the proposed classifier selection method, the accuracy was 93.08% and the recall rate was 92.78% with the top 15 base classifiers involved. A similar situation can be observed when 5 and 10 base classifiers were combined. In addition, it can also be found that the number of base classifiers was not the only factor that determined the performance of the multiclassifier system. For example, the performance of the multiclassifier system composed of all 20 base classifiers was not optimal. Moreover, in both the random selection and proposed selection methods, the performance of the multiclassifier obtained by fusing 15 base classifiers was not as good as that when fusing 10 base classifiers. These can be explained considering two facts: the random selection of the base classifier does not take into account the diversity and performance between the base classifiers. There may be redundancy between some base classifiers (e.g., training sets are similar) or data imbalances (e.g., training data are biased toward one category). These cause the classifier selection to perform better than the full ensemble.

**Figure 13:**
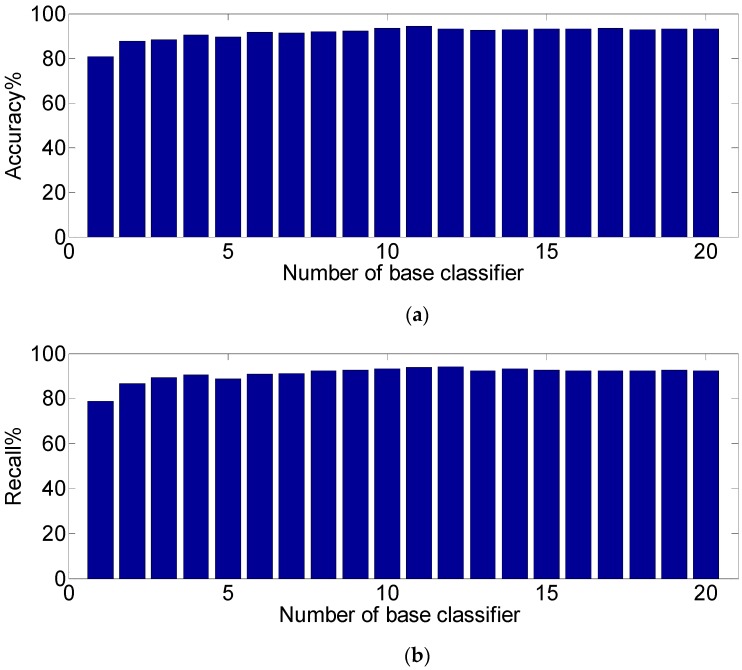
Performance of the multiclassifier system under different numbers of base classifiers: (**a**) accuracy of the multiclassifier system and (**b**) recall of the multiclassifier system.

In order to better demonstrate the advantages of using the proposed method to rank and fuse the base classifiers, we tested the performance of the multiclassifier recognition system composed of different numbers of base classifiers according to the ranking results of the proposed method. Figure 13 shows the accuracy and recall values of the recognition system with different numbers of base classifiers under the test dataset. In Figure 13, the first bar in each figure represents the 20 base classifiers with the highest classifier accuracy using the test data, and the second bar represents that the first 2 base classifiers were combined according to the ranking result of Table 7, and the last (20th) bar means that all 20 base classifiers were combined.

It can be seen from Figure 13 that with the combination of more base classifiers, the performance index of the recognition model gradually increased. When the number of combined base classifiers was 11, the two performance indexes were at the optimal values, with an accuracy of 94.29% and a recall of 94.15%. After that, as the number of base classifiers increased, the two performance indicators gradually reduced and converged to a stable value. This may be due to the fact that the base classifiers added in the top ranking had huge diversity and could form a complementary relationship with the classifier that had the highest accuracy. However, the classifiers at the bottom of the ranking may have had redundancy and reduced the overall diversity and performance of the multiclassifier system.

According to the above experimental results, the recognition system consisting of 11 base classifiers was selected for comparison with two commonly used ensemble learning methods: Bagging and AdaBoost. In addition, the base classifier with best performance and the SVM classification algorithm, which is most commonly used for activity recognition, were utilized as being representative of the single classifier. The experimental comparison results are shown in Table 9. 

It can be seen from Table 9 that the accuracy of the proposed method in the test set was 94.28%, which was 10.86, 12.43, 8.9, and 5.65 percentage points higher than the SVM, single ELM, Bagging, and AdaBoost algorithms, respectively. The recall rate of the proposed method on the test set was 93.89%, which was 10.6, 13.71, 9.17, and 6.2 percentage points higher than the SVM, single ELM, Bagging, and AdaBoost algorithms, respectively. Compared with the single ELM with the best performance and SVM, Bagging linearly combined 11 base classifiers and the recognition performance was greatly improved. This further demonstrates that multiclassifier schemes can improve the performance of HAR. However, the redundancy between the generated base classifiers of Bagging may incorporate some invalid decisions and influence the final recognition results. AdaBoost performed weighted integration of the base classifiers by assigning a large weight to the base classifier with large diversity. Therefore, the AdaBoost algorithm had better recognition performance compared with the Bagging algorithm, and the accuracy and recall rate increased by 3.25% and 2.97%, respectively. The proposed algorithm had optimal recognition performance. This may have been because it adopted an ensemble method that considered not only the accuracy but also the diversity of the base classifier; so, the multiclassifier recognition system established by the proposed method could complement the performance between the base classifiers and achieve better recognition results.

## 5. Conclusions

The inertial signals obtained by the same kind of activity under different conditions (e.g., different environments and individual differences) exhibit different characteristics. The absence of a fixed and standardized activity poses a challenge to activity recognition. In order to improve the recognition accuracy and increase the generalization performance of recognition algorithms, this paper proposed a novel activity recognition approach using a single triaxial accelerometer.

There were three critical components in our proposed approach. First, after extracting three different kinds of features from the acceleration sensor, the feature set was mapped to the new subspace by using KFDA technology to enhance the degree of discrimination of feature vectors under different activities. Second, for activity recognition, we proposed a multiclassifier system which contained ELM as the base classifier trained by the bootstrap technique. Third, the base classifiers trained by the bootstrap technique were ranked and selected based on their performance and diversity before combination. Comparative experiments with PCA- and FDA-based features showed that KFDA-based features can improve the classification accuracy effectively. In addition, it can be concluded from the experiments that the base classifiers have different structures and performances in activity recognition problems when they are trained by the bootstrap technique. Based on this, the proposed classifier selection method was utilized to optimize the classifier ensemble and showed a superior advantage compared with combining all base classifiers and the random selection method in the experiments. Apart from these comparative experiments with random selection, the proposed method also showed better performance with traditional ensemble methods, including Bagging and Adaboost.

As future work, we plan to use the EMG acquisition function of the sensor used in this paper and multisignal fusion technology to build a feature set and construct a multiclassifier recognition system with different kinds of classification algorithms. Additionally, we will attempt to use compressed sensing and deep learning methods, such as the deep belief network and the convolutional neural network, to construct two-layer diversity-enhanced activity recognition modules and engage in testing our proposed method by using datasets obtained from more body positions. 

## Figures and Tables

**Table 1:** Summary of notable activity recognition studies using wearable sensors on multiclassifier schemes and features.

Author	Year	Activities (Number Studied)	Classifier and Accuracy	Contribution
Catal [29]	2015	Walking, upstairs, downstairs, sitting, jogging, and standing (6)	Ensemble J48 decision tree, multilayer perceptron (MLP) and logistic regression (72.73%–98.7%)	Examining the power of ensemble of classifiers for activity recognition
Lee [35]	2014	Still, walk, and run (3)	Mixture-of-experts (ME) model (92.56% ± 1.05%)	The global–local cotraining algorithm was used to train the ME model
Yuan [36]	2014	Walking, running, standing, ascending and descending stairs (5)	Average combining extreme learning machine (ELM) (95.02%)	A novel ensemble learning algorithm was proposed
Cao [37]	2018	Daily and sports activities dataset (18) Opportunity (4)	ELM-based ensemble pruning for sports activities dataset (0.7848 ± 0.0077), opportunity dataset (0.9142 ± 0.0098)	Optimizing multisensor deployment by ensemble pruning
Bayat [38]	2014	Slow-walk, fast-walk, aerobic dancing, stairs-up, stairs-down (5)	MLP, LogitBoost, and SVM classifiers (91.15%)	Investigating different fusion methods to obtain an optimal set of classifiers
Ronao [40]	2016	Stand, walk, stair up, stair down, run, and lying (6)	Deep convolutional neural network; 94.79% accuracy with raw sensor data	Exploiting the inherent characteristics of activities by smartphone sensors
Khan [22]	2010	Three activity states including activities such as walking, standing, etc. (15)	Artificial neural nets (97.9%)	Linear discriminant analysis and a hierarchical approach
Hassan [41]	2018	Activities including standing, sitting, walking, lying down, stand-to-sit, etc. (12)	Deep belief network (DBN) (97.5%)	Kernel principal component analysis and linear discriminant analysis were performed to obtain more robust features
Chen [42]	2012	Daily activities including staying still, walking, running, going upstairs, and going downstairs (5)	ELM (79.68%)	Principal component analysis and ELM were utilized to realize location-adaptive activity recognition
Wang [43]	2016	Walking, upstairs, downstairs, sitting, standing, and lying (6)	k-Nearest Neighbor, KNN (87.8%) Naïve Bayes (90.1%)	Hybrid feature selection method for smart-phone-based activity recognition
Tao [44]	2016	Jumping, running, walking, step walking, walking quickly, down stairs, up stairs (7)	A new ensemble classifier termed multicolumn bidirectional long short-term memory (BLSTM); average error rates: 10.6%	Two-directional feature for BLSTM-based activity recognition
Wang [46]	2016	Standing, walking jumping, bicycling, etc. (9)	KNN with 21 features (76.42%)	Game-theory-based feature selection was used for selecting distinguished features

**Table 2:** The statistics of subjects for the experiments.

	Age	Height (cm)	Weight (kg)
Range	20–38	160–178	45–85
Mean	29.6	166	65.6
Std	6.7	5.6	13.5

**Table 3:** Activities performed in the experiments.

Activity Number	Sum (in Seconds)	Activity Number	Sum (in Seconds)
1 walk (W)	1342	5 go up stairs (GU)	1123
2 stand (S)	1253	6 sit on a chair (SC)	879
3 jump (J)	976	7 run forward (R)	1143
4 go down stairs (GD)	1034	8 lie (L)	769

**Table 4:** Confusion matrix for human activity recognition (HAR) using 20 base classifiers based on principal component analysis (PCA) features.

	W	S	J	GD	GU	SC	R	L
W	458	6	6	28	24	17	19	6
S	5	449	4	6	6	1	10	2
J	9	6	371	22	34	12	12	7
GD	31	6	17	399	5	17	11	3
GU	21	6	26	4	395	4	11	5
SC	13	3	9	10	2	432	15	3
R	15	9	13	9	14	16	441	5
L	2	1	0	0	1	3	2	350

**Table 5:** Confusion matrix for HAR using 20 base classifiers based on Fisher discriminant analysis (FDA) features.

	W	S	J	GD	GU	SC	R	L
W	528	2	3	13	8	5	2	3
S	3	458	2	3	4	3	8	2
J	2	1	447	6	8	5	2	2
GD	11	2	5	452	3	10	5	1
GU	14	7	9	2	432	1	6	1
SC	9	4	4	12	2	442	9	5
R	6	8	2	4	7	14	476	5
L	1	1	1	0	1	1	2	352

**Table 6:** Confusion matrix for HAR using 20 base classifiers based on KFDA features.

	W	S	J	GD	GU	SC	R	L
W	531	2	3	10	8	5	2	3
S	3	461	2	3	4	2	6	2
J	2	1	450	5	6	5	2	2
GD	10	2	4	456	3	9	4	1
GU	12	5	8	2	439	1	5	0
SC	8	3	3	12	1	446	9	5
R	5	9	3	2	6	12	480	5
L	1	1	1	0	1	1	1	353

**Table 7:** Diversity values for base classifiers in the multiclassifier system.

**Classifier**	**C1**	**C2**	**C3**	**C4**	**C5**	**C6**	**C7**	**C8**	**C9**	**C10**
Diversity	0.634	0.275	0.876	0.403	0.852	0.605	0.247	0	0.284	0.786
Ranking	8	19	4	13	5	9	20	1	18	6
**Classifier**	**C11**	**C12**	**C13**	**C14**	**C15**	**C16**	**C17**	**C18**	**C19**	**C20**
Diversity	0.389	0.685	0.372	0.968	0.417	0.322	0.587	0.914	0.462	0.303
Ranking	14	7	15	2	12	16	10	3	11	17

**Table 8:** The performance of fusion for all and selection of 15, 10, and 5 classifiers.

Combination Rule	Nr Classifiers	Accuracy%	Recall%
Fusion	20	93.15	92.35
Selection	15	93.08	92.78
Selection	10	93.37	93.17
Selection	5	89.68	88.68
Random	15	84.68	84.32
Random	10	85.56	84.47
Random	5	82.43	81.67

**Table 9:** Recognition performance comparison of different methods.

Method	Best Base ELM	SVM	Bagging	Adaboost	Proposed Method
Number of classifiers	1	1	11	11	11
Accuracy %	81.85	83.42	85.38	88.63	94.28
Recall %	80.18	83.29	84.72	87.69	93.89
